# Visual DNA diagnosis of *Tomato yellow leaf curl virus* with integrated recombinase polymerase amplification and a gold-nanoparticle probe

**DOI:** 10.1038/s41598-019-51650-7

**Published:** 2019-10-22

**Authors:** Tzu-Ming Wang, Jing-Tang Yang

**Affiliations:** Department of Mechanical Engineering, National Taiwan University, Taipei, 10617 Taiwan

**Keywords:** DNA probes, Biosensors, Field trials, Infectious-disease diagnostics

## Abstract

A visual DNA diagnosis with a rapid and simple procedure has been developed on integrating recombinase polymerase amplification (RPA) and a gold nanoparticle (AuNP) probe. The entire process is implemented in only one tube with no precision instrument and requires in total 20 min to amplify a DNA fragment with RPA and to discriminate a DNA fragment with an AuNP probe. The result in various colors is directly observable with the naked eye. Through discovering a small DNA fragment of *Tomato yellow leaf curl virus* (TYLCV), this system can detect one copy per microlitre of virus in a pure isolate of extracted DNA and can readily identify an infected plant with a healthy appearance. This system hence provides a highly sensitive and stable DNA diagnosis. This visual method has a potential for disease diagnosis and prognostication in the field based on advantages of simplicity, high speed, portability and sensitivity.

## Introduction

Visual observations of disease symptoms in plant tissues contribute towards the diagnosis of disease, but this approach typically requires an experienced pathologist and is unreliable when symptoms appear at an early stage of infection. Early detection of plant pathogens is important in the management of plant disease, especially for plant quarantine at a country border and seedling cultivation in a greenhouse^1^. Many molecular biotechnological approaches have been widely applied to diagnose plant disease including immunology-based and nucleic-acid-based methods, which investigate pathogen proteins and nucleic acids extracted from plant materials and can substitute for the observation and cultivation of pathogens^2^. Immunology-based methods, such as enzyme-linked immunosorbent assay (ELISA) and lateral flow devices (LF), are useful to detect the pathogen antigens and have been developed into portable equipment. As the specificity of these methods has relied on monoclonal antibodies, the sensitivity generally varies with the species^3^. Nucleic-acid-based methods involve mainly a polymerase chain reaction (PCR), which first amplifies a specific gene or DNA fragment for subsequent detection with gel electrophoresis. Basic and advanced PCR methods are at present widely used to detect plant pathogens because of their great sensitivity^2,4,5^. Nucleic-acid-based methods have greater accuracy, specificity and sensitivity than methods based on immunology, but they rely strongly on expertise and instruments in the laboratory, which becomes a limitation for their portable application in the field^6,7^. To overcome these limitations, prominent and portable isothermal nucleic-acid amplification techniques and biosensors provide a solution to the drawbacks of their counterparts, respectively.

Isothermal amplification techniques have recently been employed for genotyping, disease diagnosis, whole genome amplification, miniaturization and automation of bioanalytical applications based on their isothermal properties, simple procedures and excellent amplification capabilities. Recombinase polymerase amplification (RPA) can work satisfactorily at constant temperatures ranging from 25 to 45 °C^8–10^, and even using human body heat^11^. The reaction is complete in 20–40 min but the duration of detection limit is as little as 5 min. Among those approaches, RPA has the advantages of being simple and convenient to perform at a low temperature and for a short period while still possessing the characteristics of exponential amplification efficiency and highly sensitive detection^12^. The applications of RPA in the diagnosis of plant pathogens have been reported, including *Tomato yellow leaf curl virus* (TYLCV)^13–20^. The RPA amplicons are unsuitable for detection with agarose gel electrophoresis and should be purified with PCR cleanup columns. Specially designed probes, enzymes, lateral flow strips and further processing are involved in the detection of RPA amplicons^8,21^. A simple and effective detection of RPA amplicons is the next issue in the development of rapid and sensitive diagnostic techniques.

Innovative and portable DNA-based biosensors have been used as diagnostic tools in recent years; they exhibit rapid and sensitive characteristics and can allow qualitative and quantitative analysis of pathogens with label-free techniques. For reasons of simplicity and convenience, a gold nanoparticle (AuNP)-labeled DNA probe as an optical DNA sensor is the best method for DNA detection^7^. A colorimetric detection of a AuNP probe recognized with amplicons is implemented mostly with a lateral flow strip^22,23^ and AuNP aggregation^24–26^. Although no additional equipment is required to detect AuNP aggregation with the naked eye, there are still shortcomings such as accuracy, sensitivity and quantitative interpretation. A novel approach can enhance the reliability of colorimetric detection of AuNP aggregation in qualitative and quantitative analysis with the naked eye^27^, which can facilitate an expansion of the applications of a AuNP probe in DNA detection.

With the aim of accelerating the reaction and simplifying the procedure, we have integrated RPA with an optical AuNP probe to detect pathogen DNA with the naked eye. All reaction conditions in the procedure of a visual DNA diagnostic technique have been completely optimized under a modest requirement of equipment. In this work TYLCV was chosen as a model pathogen because it can infect tomato and genus *Solanum* plants, and cause serious economic losses^28^. The performance of our approach is compared directly with conventional bench-top methods. This rapid and simple system can be employed in qualitative and quantitative analysis of pathogens and broad applications in disease diagnosis and prognostication.

## Materials and Methods

### Sample preparation and DNA extraction

Healthy tomatoes were planted in an insect-free net house and then placed with TYLCV-viruliferous whitefly (*Bemisia tabaci* biotype B) for one week. The viral DNA was extracted from leaves of TYLCV-infected tomato plants using a Gene-Spin^TM^ GenomicDNA Isolation Kit, Protech Technology Enterprise Co., Taipei, Taiwan). The plasmid DNA was extracted from liquid-cultured *Escherichia coli* (TOP10, Thermo Fisher Scientific Inc., MA, USA) carrying TYLCV isolate 82-2-1 and 57-2 clones.

### Primer design and conventional PCR analysis

The nucleotide sequences of 56 Taiwan TYLCV isolates adapted from Tsai *et al*.^29^ were applied in a multiple sequence alignment using software (JalView, version 2.10.5)^30^. According to the consensus of multiple sequences, the TYLCV- specific primer set 1/2R (5′-GGATTAGAGGCATGAGTACA-3′/5′-GGATTAGAGGCATGAGTACATGCCATATA-3′) was designed (Supplementary Fig. S1) and synthesized (Mission Biotech, Taipei, Taiwan). A conventional PCR reaction was performed in total volume 10 μL containing isolated DNA (5 ng), 1x Taq DNA Polymerase Master Mix RED (Ampligon, Odense, Denmark) and a TYLCV-specific primer set 1/2R (0.1 μM), and then implemented in a thermal cycler (Labcycler, SensoQuest, Göttingen, Germany) using this program: denaturation at 95 °C for 5 min followed by 30 cycles of 95 °C for 30 s, 55 °C for 30 s, and 72 °C for 30 s and final extension at 72 °C for 10 min. To assess the sensitivity, we applied successively varied DNA concentrations of TYLCV isolate 82-2-1 in the PCR reaction from 10^9^ to 1 copies per μL. The amplicon (10 μL) was eventually validated with gel electrophoresis on agarose gel (1.5%).

### Sequence analysis and gold nanoparticle (AuNP) probe design

**Figure 1:**
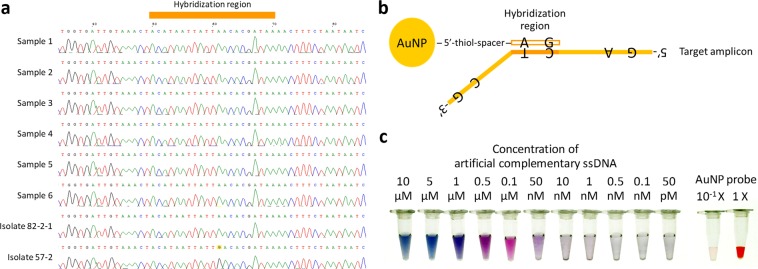
AuNP probe design. (**a**) Sequences of target fragment amplified from various TYCLV-infected plants and reference isolates. Isolate 57-2 carried a single nucleotide polymorphism (SNP) (A/G) at base 61. (**b**) Schematic of DNA colorimetric diagnosis through an AuNP probe hybridized with complementary target amplicon. (**c**) Sensitivity test of AuNP probe hybridized with specific-artificial cDNA.

Conventional PCR amplicons were ligated into a vector (pCR™ 2.1-TOPO^®^, TOPO^®^ TA Cloning^®^ Kit; Thermo Fisher Scientific Inc., MA, USA) and sequenced (Mission Biotech, Taipei, Taiwan). The highly conserved sequence of conventional PCR amplicons derived from separate samples and TYLCV isolates was discovered and designed to be the probe DNA conjugated with AuNP (Fig. 1a). For the synthesis of the AuNP probe, the probe DNA was modified with the 5′-end labeled thiol (C6SH) (5′-thiol-TATCGTGTTAATAATTATGT-3′). Artificial cDNA complementary to probe DNA was also designed (5′-ATGGTTCTCGTACTTCCCAGCTTCCTGGTGATTGTAAACTACATAATTATTAACACGATA-3′) (Mission Biotech, Taipei, Taiwan) (Supplementary Fig. S1).

### AuNP probe preparation and colorimetric assay

The AuNP solution (particle size 13 nm) was purchased (TANBead^®^ NanoGold-13, Taiwan Advanced Nanotech Inc., Taipei, Taiwan). The AuNP probe was prepared according to the literature procedure^27^. The concentration of the AuNP probe was growth-regulated to 50 nM with pure water and stored at 4 °C for ensuing use. For colorimetric assay, the AuNP probe (1 μL, 50 nM) was added into DNA solution (100 μL) with sodium chloride (15 μL, 3 M). Artificial complementary ssDNA solution was examined at varied concentrations of from 50 pM to 10 μM. The admixture of the DNA sample and the AuNP probe was incubated at 95 °C for 5 min. Hydroxylamine hydrochloride (H_2_NOH, 1.5 μL, 400 mM) and hydrogen tetrachloroaurate (III) (HAuCl_4_, 1.5 μL, 25 mM, Sigma-Aldrich, USA) were subsequently added and mixed with the solution. After approximately 30 s, the AuNP was grown and visualized with various colors. UV/visible spectral analysis of the growth AuNP solution was implemented with a spectrophotometer (NanoDrop^TM^ 2000, Thermo Fisher Scientific Inc., MA, USA).

### Recombinase polymerase amplification (RPA) and AuNP colorimetric assay

The recombinase polymerase amplification (RPA) reaction was achieved using a basic kit (TwistAmp^®^, TwistDX, Cambridge, UK) according to the manufacturer’s recommendations with some modifications. RPA reactions were performed in total volume 20 μL containing isolated DNA (5 ng) and primer set (480 nM) and then implemented at 39 °C for 30 min. To establish the optimal conditions for DNA detection, RPA reactions were executed at successively 33, 35, 37 and 39 °C for 5, 10, 20 and 30 min. To test the sensitivity, various DNA concentrations of TYLCV isolate 82-2-1 were applied in the RPA reaction from 10^9^ to 1 copies per μL in an appropriate order, and were then incubated at 33 °C for 10 min. RPA amplicons (4 μL) were validated with gel electrophoresis on agarose gel (1.5%). RPA amplicons (10 μL) were used in a AuNP colorimetric assay. For visual DNA diagnosis, a AuNP probe (50 nM, 1 μL for testing RPA amplicons, 3.5 μL for sensitivity study of visual DNA diagnosis) was added into RPA amplicon (10 μL) with blending and then incubated at 95 °C for 5 min. Subsequently, H_2_NOH (1 μL, 200 mM) and HAuCl_4_ (1 μL, 12.5 mM) were added and mixed. After approximately 30 s, the AuNP was grown and visualized with various colors. The image and UV/visible spectra of the growth AuNP solution were finally recorded.

### Gel image analysis

All gel images were acquired with a digital gel image system (DigiGel, DGIS-10c, TOPBIO Co., New Taipei City, Taiwan) and recorded in JPEG format. The gel images were processed with cropping, automatic adjustment of brightness and contrast, and quantification of electrophoretic bands using Fiji (image analysis software)^31^. Signal values with 4 replicates are expressed as an arithmetic mean ± standard deviation (s.d.). The correlation between amount of amplicons and template concentration was determined with linear regression using Microsoft Excel 2016.

## Results and Discussion

### Design concept of visual DNA diagnosis

**Figure 2:**
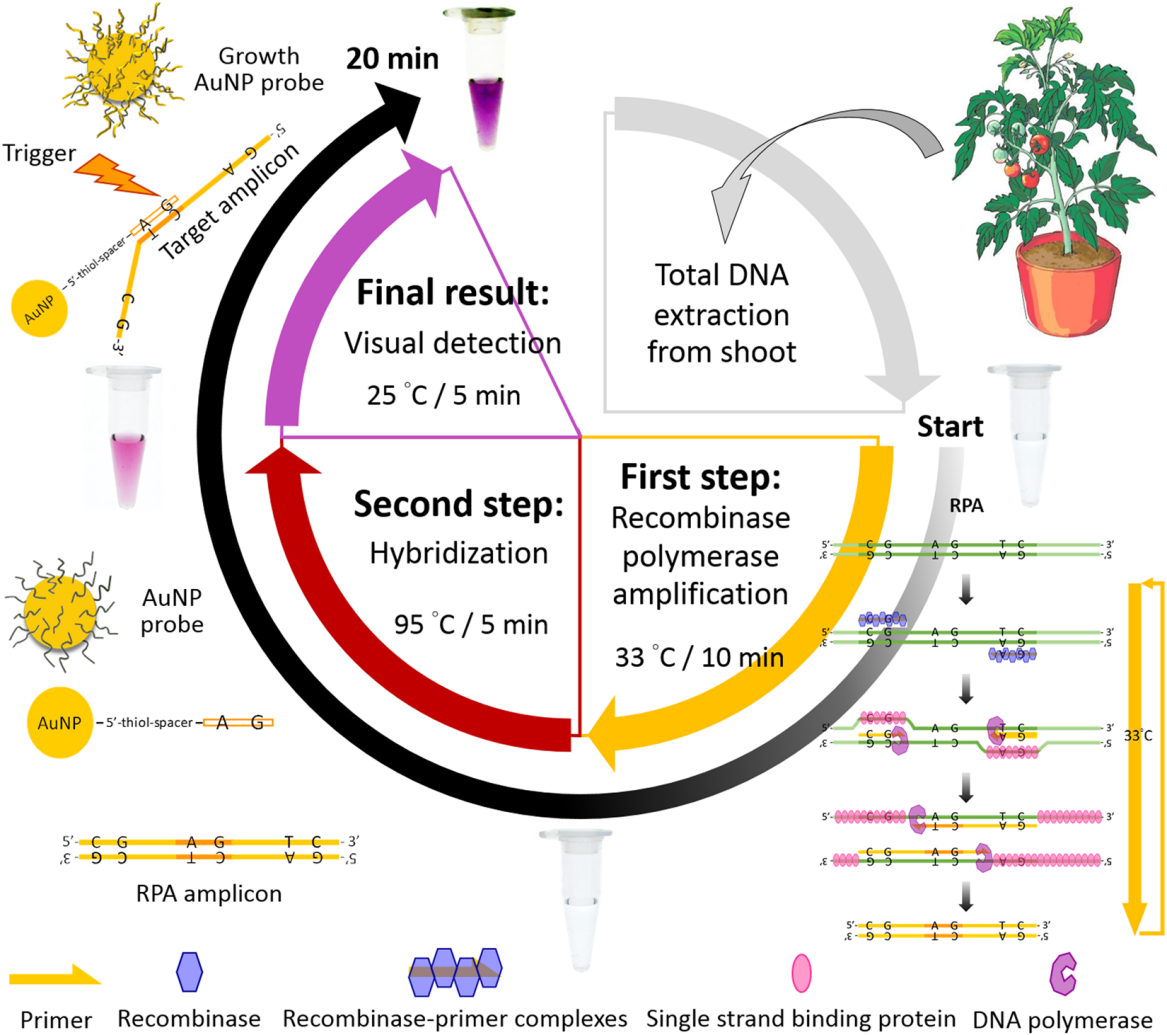
Schematic illustration of visual DNA diagnosis of TYLCV in one tube.

To clarify the entire concept of visual TYLCV DNA diagnosis in one tube, the procedure is described with a schematic illustration (Fig. 2). Apart from a total DNA extraction, two main steps were conducted in three stages in visual DNA diagnosis, including RPA, hybridization of AuNP probe and visual detection. In the first step, extracted total DNA (1 μL) was added into RPA solution (9 μL) containing a specific primer set and then incubated at 33 °C for 10 min to amplify the TYLCV-specific DNA fragment. The specific primer set aids the determination of the presence and types of pathogens. The RPA reaction works as well as conventional PCR at a constant low temperature and requires less duration^8–10,32^. This approach achieves saving of time and effort, with modest requirement of equipment and expertise in DNA amplification using RPA.

In the second step, AuNP probe (3.5 μL) was added directly to the RPA reaction (10 μL) with rigorous blending. For the purpose of highly stringent hybridization between a AuNP probe and a TYLCV-specific DNA fragment, the admixture was incubated at 95 °C for 5 min. Immediately afterward, H_2_NOH and HAuCl_4_ (1 μL) were individually added and blended to trigger the growth of AuNP. At 95 °C, the amplified DNA fragments were denatured and transformed into ssDNA. Because a DNA probe conjugated with AuNP hybrids only complementary ssDNA and develops a double helix, a AuNP probe can discriminate complementary ssDNA from mismatched ssDNA. Moreover, the amount and sequence similarity of cDNA would affect the growth, size and shape of AuNP; the sizes and shapes of AuNP have varied colors and UV/visible spectra^33^. The color variation of growth AuNP can hence be regarded as a semi-quantitative interpretation of the cDNA concentration^27^. This feature shows that the detection of DNA relying on the AuNP probe can be accomplished with the naked eye. RPA integrated with a AuNP probe can fulfill a simple and convenient DNA diagnosis. In the future, this system could be applied to diagnosis of disease in the field.

### Colorimetric assay of TYLCV-identified AuNP probe

The design of an identified amplicon and AuNP probe is a priority in visual DNA diagnosis. To fulfill the requirement of RPA reactions, the newly TYLCV-identified primer set was redesigned and carried a smaller length, greater sensitivity and greater accuracy than the conventional primer set (Supplementary Fig. S2). According to the nucleotide sequences amplified from separate infected samples and TYLCV isolates, a DNA probe conjugated with AuNP was designed (Fig. 1a). Artificial complementary ssDNA was also designed the same as a TYLCV-identified amplicon that can hybridize with a AuNP probe (Fig. 1b).

To clarify the effectiveness and sensitivity of the AuNP-probe detected target amplicon, cDNA of varied concentration was successively hybridized with a AuNP probe. The color of the growth AuNP altered from pink to other colors in a sequence,–dark blue, navy blue, indigo, purple, purple-red, mauve, which characterize cDNA concentrations 10, 5, 1, 0.5, 0.1 μM, 50 nM, respectively. A concentration less than 10 nM became an indistinct color; even a precipitate appeared at less than 0.5-nM reaction (Fig. 1c). Colorimetric assay of TYLCV-identified AuNP is hence feasible; the color variation of the solutions provides a semi-quantitative interpretation of the cDNA concentration. Unlike in previous work^27^, this assay used long cDNA (60 nt) to hybridize with a short AuNP probe (20 nt). The increased length of cDNA meets the requirement of DNA identification. A longer cDNA seems to generate a wide range of color variation of growth AuNP solutions, suitable for visual observation because of an improved linear distribution of colors and cDNA concentrations.

### Optimization of the RPA condition

**Figure 3:**
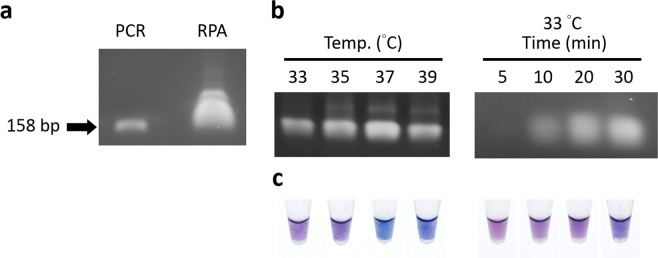
Optimization of RPA conditions. (**a**) Comparison of products of conventional PCR and RPA. (**b**) Amplification of varied RPA temperature for 30 min and varied RPA durations at 33 °C. (**c**) Colorimetric detection at varied RPA temperature for 30 min and varied RPA duration at 33 °C. Full-length gels are presented in Supplementary Fig. S4.

In an optimal condition, the RPA reactions (TwistAmp^®^ Basic Kit) work at a constant temperature (37–39 °C) for 20–40 min based on the manufacturer’s recommendations. The yield of RPA amplicons generated at 39 °C for 40 min is more than that of conventional PCR (Fig. 3a). The RPA reaction can operate satisfactorily in a temperature range 25–45 °C^9,10^, especially at human body heat (33 °C)^11^. The reaction can be detected in only 5 min^8,10,32^. To save time and effort, we studied the best conditions of RPA reactions to detect TYLCV. RPA reactions were executed successively at 33, 35, 37 and 39 °C for 5, 10, 20 and 30 min. As the result of gel electrophoresis, RPA amplicons had a maximum yield at 37 °C and a minimum yield at 33 °C for 30 min. The effect of varied duration indicates that a low limit of detection was generated at 10 min (Fig. 3b).

The colorimetric detection was subsequently examined to understand the feasibility of a low detectable condition appropriate to a RPA reaction integrated with a AuNP probe. The colors of growth AuNP hybridized with various RPA reactions were purple, blue-violet, navy blue, which characterize reaction temperatures from 33, 35, 37 to 39 °C, respectively. Even the color of slight (10 min) and indistinct (5 min) detectability of RPA reactions were both purple. The color of a AuNP probe detected in various RPA reactions is similar to various concentrations of artificial complementary ssDNA (Fig. 3c). These results reveal that the detectably efficient condition for the RPA reaction is incubation at 33 °C for 10 min, which can be applied to DNA detection with a AuNP probe.

### Sensitivity of visual DNA diagnosis

**Figure 4:**
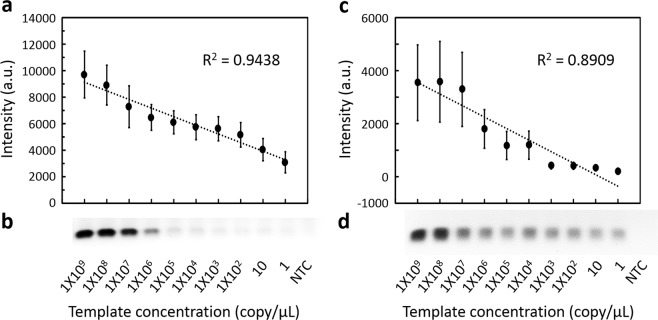
Sensitivity test of TYLCV DNA diagnosis through conventional PCR and RPA. (**a**) Plot of correlation between amount of PCR amplicons and template concentration. (**b**) Gel electrophoresis image of PCR. (**c**) Plot of correlation between amount of RPA amplicons and template concentration. (**d**) Gel electrophoresis image of RPA. NTC, no template control. Error bars represent ± s.d., *n* = 4. Full-length gels are presented in Supplementary Fig. S5.

To comprehend the limit of a successful procedure of visual DNA diagnosis, we examined the sensitivity of TYLCV DNA diagnosis. The sensitivity of RPA was compared to conventional PCR using the same varied amounts of TYLCV isolate 82-2-1 DNA. Based on gel electrophoresis, there were positive correlations between amplification effect and template concentration in both conventional PCR (*R*^2^ = 0.9438) and RPA (*R*^2^ = 0.8909). RPA (1 copy/μL) was clearly one million times as sensitive as conventional PCR (10^6^ copies/μL) (Fig. 4) and more rapid than quantitative real-time PCR (qPCR) (Supplementary Fig. S3).

**Figure 5:**
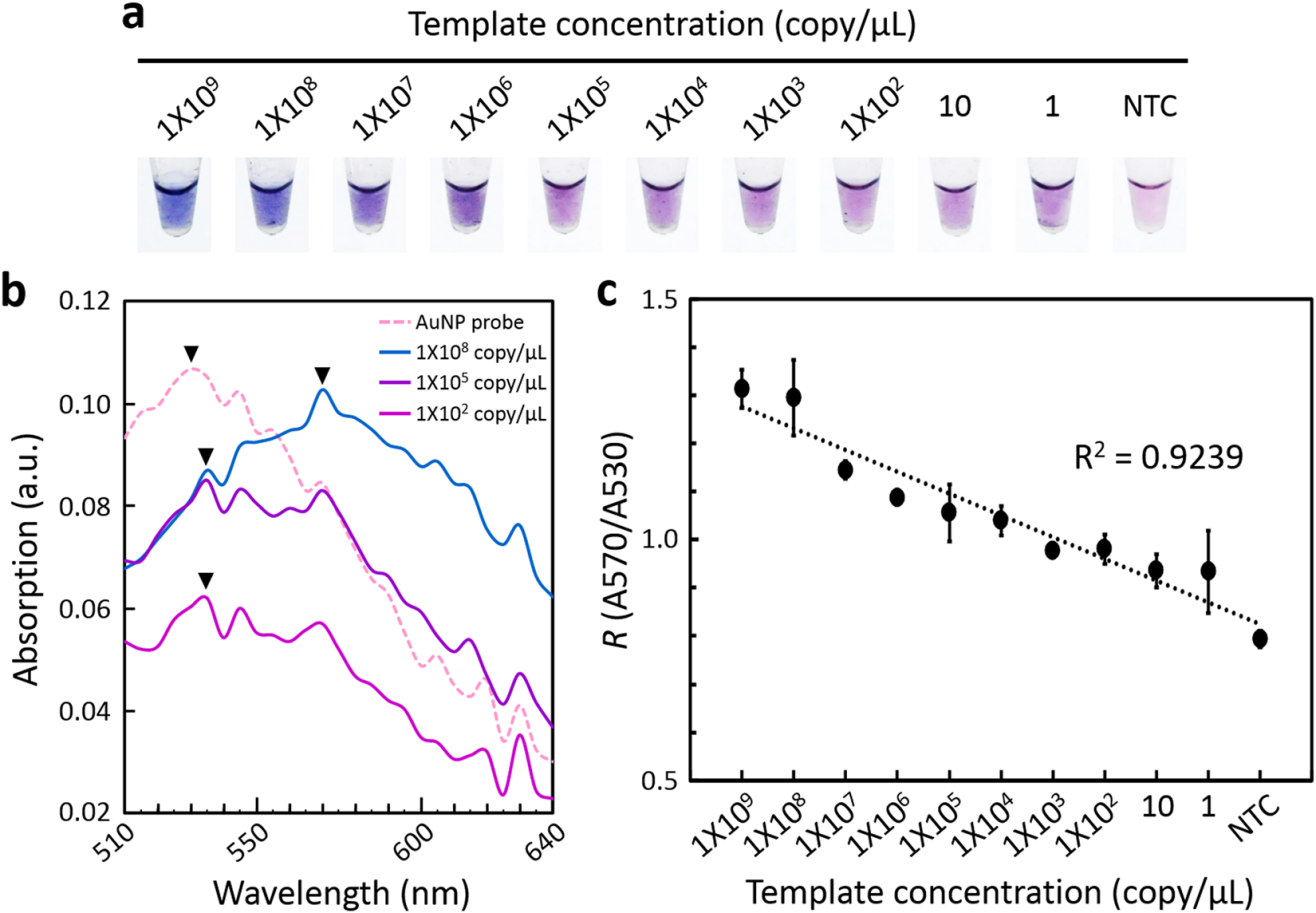
Sensitivity test of visual DNA diagnosis using a reference isolate. (**a**) Colorimetric detection and (**b**) Absorption spectra of growth AuNP solutions derived from RPA amplicons. ▼ indicates the absorption maximum of each growth AuNP solution. (**c**) Plot of correlation between absorption ratio *R* (A570/A530) and template concentration. NTC, no template control. Error bars represent ± s.d., *n* = 4.

Afterward, the sensitivity of AuNP-probe-detected RPA amplicons was realized on discovering the color and UV/visible spectra of growth AuNP solutions. The colors derived from RPA amplicons over a range of template concentrations altered obviously from pink (NTC) to colors in a sequence, such as mauve (1–10^3^ copies/μL), purple (10^4^–10^5^ copies/μL), blue-violet (10^6^–10^7^ copies/μL) and navy blue (10^8^–10^9^ copies/μL) (Fig. 5a). The absorption maximum of growth AuNP that altered from pink (530 nm, A530) to blue (570 nm, A570) (Fig. 5b) is a phenomenon of a blue shift (Fang *et al*.^27^). To clarify accurately the relation between color and template concentration, an absorption ratio *R* (A570/A530) of growth AuNP was estimated. The relation between absorption ratio *R* and template concentration showed a linear distribution (*R*^2^ = 0.9239) (Fig. 5c). It is significant that 1 copy/μL of TYLCV can be detected with visual DNA diagnosis, whereas the amount of virus that reached 10^6^ copies/μL showed a rich color.

### Reliability assessment of visual DNA diagnosis

**Figure 6:**
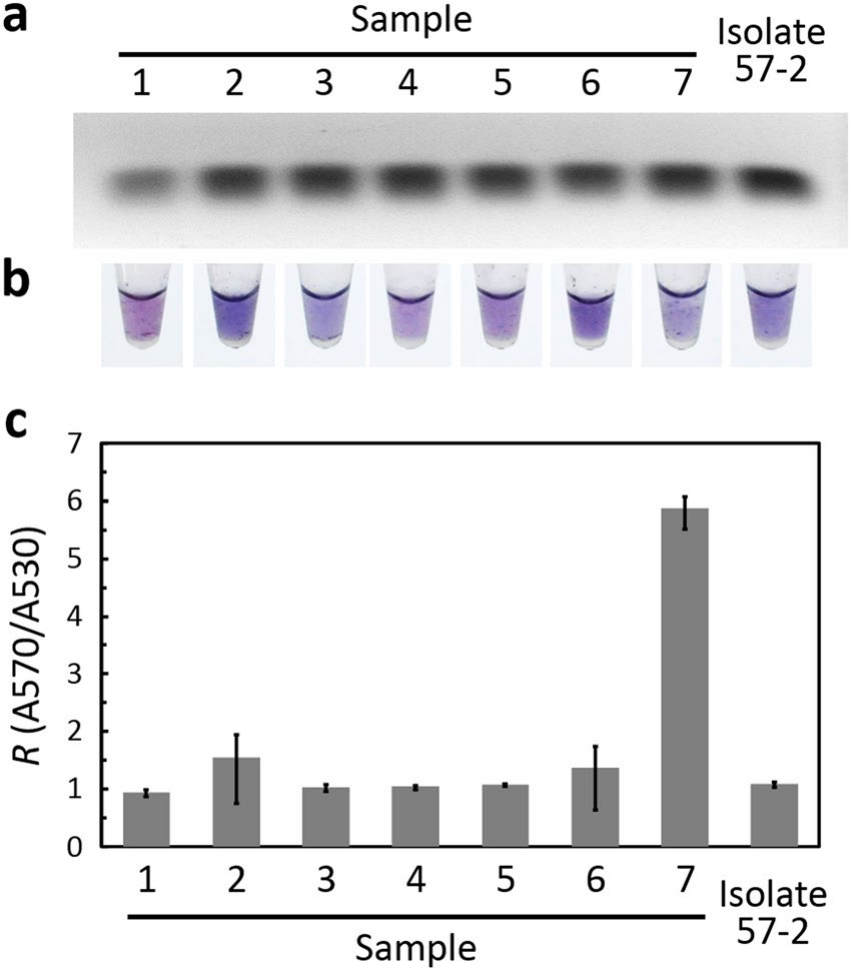
Reliability assessment of visual DNA diagnosis using infected plants. (**a**) Gel electrophoresis image of RPA, (**b**) Colorimetric detection, and (**c**) Absorption ratio *R* (A570/A530) of growth AuNP solutions derived from TYLCV-infected plants and isolate 57-2. Error bars represent ± s.d., *n* = 4. Sample 1 had a healthy appearance. Samples 2–7 were severely damaged. Absorption ratio *R* of NTC = 0.79. Full-length gels are presented in Supplementary Fig. S6.

The nucleic-acid extraction of pathogens is invariably accompanied with a large amount of host genomic DNA (gDNA), because the nucleic-acid concentration of a pathogen is diluted with many host gDNA, which would interfere with the DNA diagnosis. For this reason, the reliability of visual DNA diagnosis was verified by means of infected tomato plants with various symptoms. Seven infected samples were diagnosed with conventional PCR (Supplementary Fig. S2b,c) and nucleotide sequencing (Fig. 1a). The RPA reactions of those infected plants were confirmed with gel electrophoresis, colorimetric assay and absorption ratio *R* (A570/A530), respectively (Fig. 6). The results of gel electrophoresis showed the virus amount of the samples, but there was no significant difference among samples with a large amount of virus. The AuNP color of sample 1 with a healthy appearance showed purple whereas severely damaged samples 2–7 and reference isolate 57-2 showed purple-blue or blue-violet. The absorption ratio *R* (A570/A530) definitely indicated that sample 1 (*R* = 0.95) carried 10 copies/μL (*R* = 0.94) of TYLCV; the virus amount of sample 2 (*R* = 1.55) was more than 10^9^ copies/μL (*R* = 1.31). Samples with a large amount of virus were expected to show a rich color, *i.e*. blue or blue-violet, and a large absorption ratio *R* (>1), except sample 7 that had an extremely large absorption ratio possibly due to the precipitation of AuNP. Moreover, reference isolate 57-2 with single nucleotide polymorphism (SNP) (Fig. 1a) still showed a poor performance in color and absorption ratio *R* under a large template concentration (10^9^ copies/μL). This phenomenon was revealed by Fang *et al*.^27^, but the diagnostic result retains discrimination. In general, the diagnostic results of the colorimetric assay and the absorption ratio were consistent; visual DNA diagnosis can hence be applied to TYLCV identification.

## Conclusion

We developed a visual DNA diagnosis with integrated RPA and a AuNP probe. This work demonstrates that this excellent system can amplify and detect DNA in a rapid and simple procedure and maintain a high sensitivity. DNA amplified with RPA at a low temperature for less duration achieves saving of time and effort. RPA amplicons discriminated with a biosensor AuNP probe form a visible solution of varied color, *i.e*. mauve, purple, blue-violet, navy blue. The entire procedure consumed only 20 min and a modest requirement of equipment. According to the sensitivity test of this visual TYLCV DNA diagnosis, the results showed that this system is more rapid and more sensitive than conventional PCR and qPCR. In particular, 1 copy/μL of the virus and an infected plant with a healthy appearance can still be identified. The colors of the reactions can process the qualitative and quantitative analysis of virus. This system can clearly detect the virus in infected tomato plants even with an interference from plant genomic DNA in accordance with the reliability assessment. Based on the advantages of the above description, this system can be applied directly to monitoring health in the field. As the procedure and reagents of this system are simple and convenient, it could be combined with a microfluidic system to accomplish a multiplex or singleplex diagnosis.

## Supplementary information

Supplementary information

## References

[1] Miller SA, Beed FD, Harmon CL (2009). Plant disease diagnostic capabilities and networks. Annual Review of Phytopathology.

[2] López MM (2003). Innovative tools for detection of plant pathogenic viruses and bacteria. International microbiology: the official journal of the Spanish Society for Microbiology.

[3] Nolasco G (2002). Asymmetric PCR ELISA: increased sensitivity and reduced costs for the detection of plant viruses. European Journal of Plant Pathology.

[4] Schaad NW, Frederick RD (2002). Real-time PCR and its application for rapid plant disease diagnostics. Canadian Journal of Plant Pathology.

[5] Lievens B, Brouwer M, Vanachter ACRC, Cammue BPA, Thomma BPHJ (2006). Real-time PCR for detection and quantification of fungal and oomycete tomato pathogens in plant and soil samples. Plant Science.

[6] Fang Y, Ramasamy RP (2015). Current and Prospective Methods for Plant Disease Detection. Biosensors.

[7] Khater M, de la Escosura-Muñiz A, Merkoçi A (2017). Biosensors for plant pathogen detection. Biosensors and Bioelectronics.

[8] Piepenburg O, Williams CH, Stemple DL, Armes NA (2006). DNA detection using recombination proteins. PLoS Biology.

[9] Sun K (2016). Recombinase polymerase amplification combined with a lateral flow dipstick for rapid and visual detection of *Schistosoma japonicum*. Parasites and Vectors.

[10] Lillis L (2014). Non-instrumented incubation of a recombinase polymerase amplification assay for the rapid and sensitive detection of proviral HIV-1 DNA. PLoS One.

[11] Crannell ZA, Rohrman B, Richards-Kortum R (2014). Equipment-free incubation of recombinase polymerase amplification reactions using body heat. PLoS One.

[12] Deng H, Gao Z (2015). Bioanalytical applications of isothermal nucleic acid amplification techniques. Analytica Chimica Acta.

[13] Mekuria TA, Zhang S, Eastwell KC (2014). Rapid and sensitive detection of Little cherry virus 2 using isothermal reverse transcription-recombinase polymerase amplification. Journal of Virological Methods.

[14] Zhang S (2014). Rapid diagnostic detection of plum pox virus in *Prunus* plants by isothermal AmplifyRP® using reverse transcription-recombinase polymerase amplification. Journal of Virological Methods.

[15] Glais, L. & Jacquot, E. In *Plant Pathology* Vol. 1302 *Methods in Molecular Biology* (ed. Lacomme C.) Ch. 16, 207–225 (Humana Press, 2015).

[16] Silva G, Bomer M, Nkere C, Kumar PL, Seal SE (2015). Rapid and specific detection of *Yam mosaic virus* by reverse-transcription recombinase polymerase amplification. Journal of Virological Methods.

[17] Londoño MA, Harmon CL, Polston JE (2016). Evaluation of recombinase polymerase amplification for detection of begomoviruses by plant diagnostic clinics. Virology Journal.

[18] Babu B (2017). A field based detection method for *Rose rosette virus* using isothermal probe-based Reverse transcription-recombinase polymerase amplification assay. Journal of Virological Methods.

[19] Babu B (2017). A rapid assay for detection of *Rose rosette virus* using reverse transcription-recombinase polymerase amplification using multiple gene targets. Journal of Virological Methods.

[20] Kapoor R (2017). Development of a recombinase polymerase amplification assay for the diagnosis of banana bunchy top virus in different banana cultivars. Archives of Virology.

[21] Kersting S, Rausch V, Bier FF, von Nickisch-Rosenegk M (2014). Multiplex isothermal solid-phase recombinase polymerase amplification for the specific and fast DNA-based detection of three bacterial pathogens. Mikrochimica Acta.

[22] Zhao W (2011). Rapid on-site detection of *Acidovorax avenae* subsp. *citrulli* by gold-labeled DNA strip sensor. Biosensors and Bioelectronics.

[23] Wei J (2014). Miniaturized paper-based gene sensor for rapid and sensitive identification of contagious plant virus. ACS Applied Materials and Interfaces.

[24] Gill P, Alvandi AH, Abdul-Tehrani H, Sadeghizadeh M (2008). Colorimetric detection of *Helicobacter pylori* DNA using isothermal helicase-dependent amplification and gold nanoparticle probes. Diagnostic Microbiology and Infectious Disease.

[25] Vaseghi A, Safaie N, Bakhshinejad B, Mohsenifar A, Sadeghizadeh M (2013). Detection of *Pseudomonas syringae* pathovars by thiol-linked DNA–Gold nanoparticle probes. Sensors and Actuators B: Chemical.

[26] Xing Y (2013). A colorimetric method for H1N1 DNA detection using rolling circle amplification. The Analyst.

[27] Fang W-F, Chen W-J, Yang J-T (2014). Colorimetric determination of DNA concentration and mismatches using hybridization-mediated growth of gold nanoparticle probes. Sensors and Actuators B: Chemical.

[28] Smith HA, Seijo TE, Vallad GE, Peres NA, Druffel KL (2015). Evaluating Weeds as Hosts of *Tomato yellow leaf curl virus*. Environmental Entomology.

[29] Tsai WS, Shih SL, Kenyon L, Green SK, Jan FJ (2011). Temporal distribution and pathogenicity of the predominant tomato-infecting begomoviruses in Taiwan. Plant Pathology.

[30] Waterhouse AM, Procter JB, Martin DM, Clamp M, Barton GJ (2009). Jalview Version 2–a multiple sequence alignment editor and analysis workbench. Bioinformatics (Oxford, England).

[31] Schindelin J (2012). Fiji: an open-source platform for biological-image analysis. Nature methods.

[32] Krõlov K (2014). Sensitive and rapid detection of *Chlamydia trachomatis* by recombinase polymerase amplification directly from urine samples. The Journal of Molecular Diagnostics: JMD.

[33] Wang Z, Zhang J, Ekman JM, Kenis PJ, Lu Y (2010). DNA-mediated control of metal nanoparticle shape: one-pot synthesis and cellular uptake of highly stable and functional gold nanoflowers. Nano letters.

