# Supplementary material for: Visual DNA diagnosis of Tomato yellow leaf curl virus with integrated recombinase polymerase amplification and a gold-nanoparticle probe
Source: Sci Rep. 2019 Oct 22;9:15146. doi: 10.1038/s41598-019-51650-7 (PMC6805851; doi:10.1038/s41598-019-51650-7)
Supplement: Supplementary file 1 — Supplementary information [file 41598_2019_51650_MOESM1_ESM.pdf]

Visual DNA diagnosis of *Tomato yellow leaf curl virus* with integrated recombinase polymerase amplification and a gold-nanoparticle probe

Tzu-Ming Wang, and Jing-Tang Yang

Department of Mechanical Engineering, National Taiwan University, Taipei 10617, Taiwan

Corresponding author

Jing-Tang Yang
Department of Mechanical Engineering
National Taiwan University,
No. 1, Sec. 4, Roosevelt Rd., Taipei 10617, Taiwan (R.O.C.)

Tel: +886-2-33669875, +886-2-33669934
Fax: +886-2-33669548
E-mail: jtyang@ntu.edu.tw

Supplementary Information

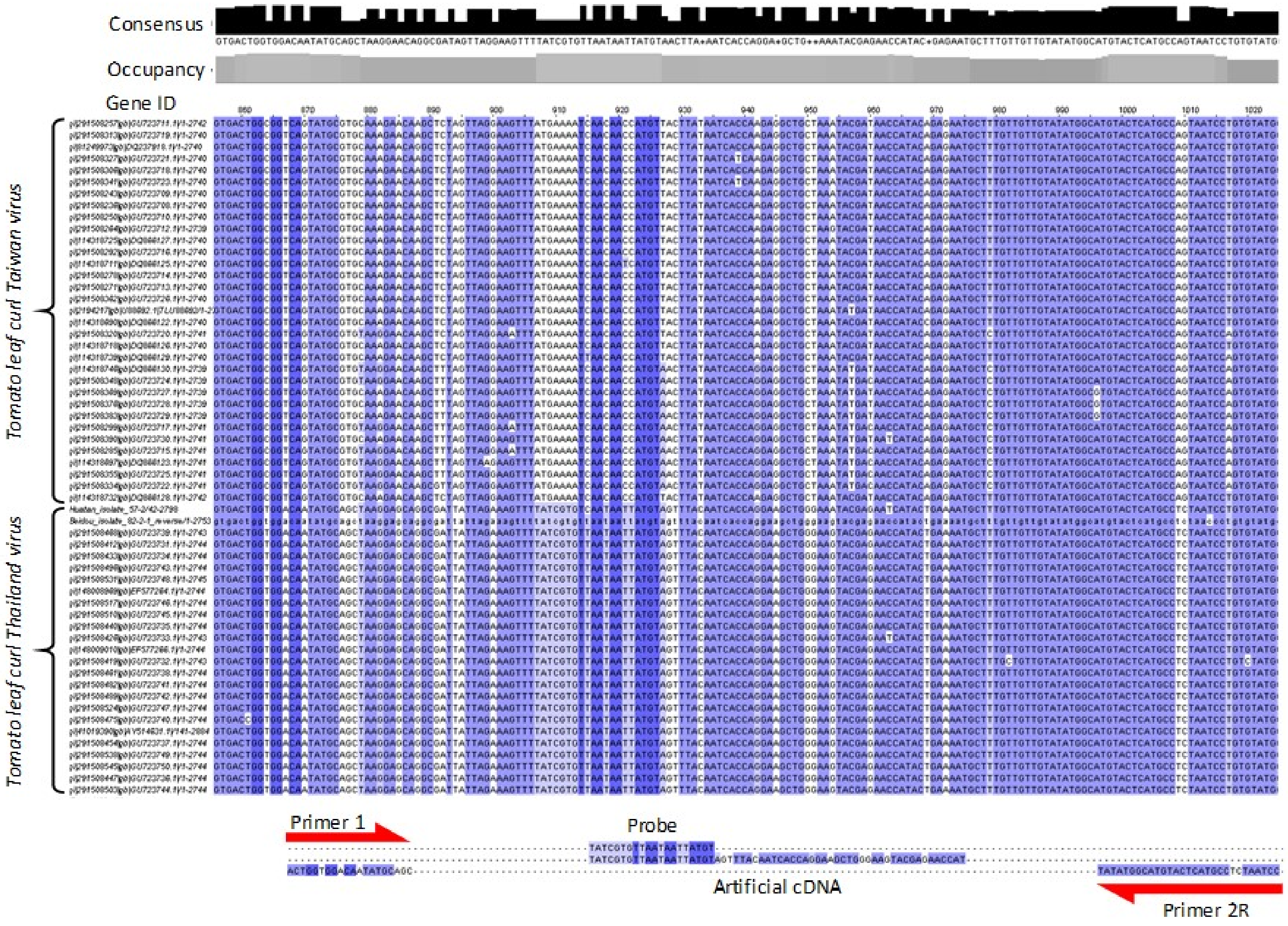

Supplementary Figure S1. Primer and AuNP probe design for TYLCV DNA diagnosis. The design of primer set 1/2R proceeded according to the alignment of 56 *Begomovirus* DNA A genome sequences, which consist of ToLCTWV (*Tomato leaf curl Taiwan virus*) and TYLCTHV (*Tomato yellow leaf curl Thailand virus*) and were adapted from Tsai *et al.* (2011). The design of a probe conjugated with AuNP and complementary DNA relied on the amplicon sequence of primer set 1/2R. The primer, probe and artificial cDNA can significantly distinguish two isolates as the designs correspond to a specific isolate. A blue background of a nucleotide represents the consensus among multiple sequences; dark blue has greater consistency, whereas white shows inconsistency.

Tsai, W. S., Shih, S. L., Kenyon, L., Green, S. K. & Jan, F. J. Temporal distribution and pathogenicity of the predominant tomato-infecting begomoviruses in Taiwan. *Plant Pathology* **60**, 787-799 (2011).

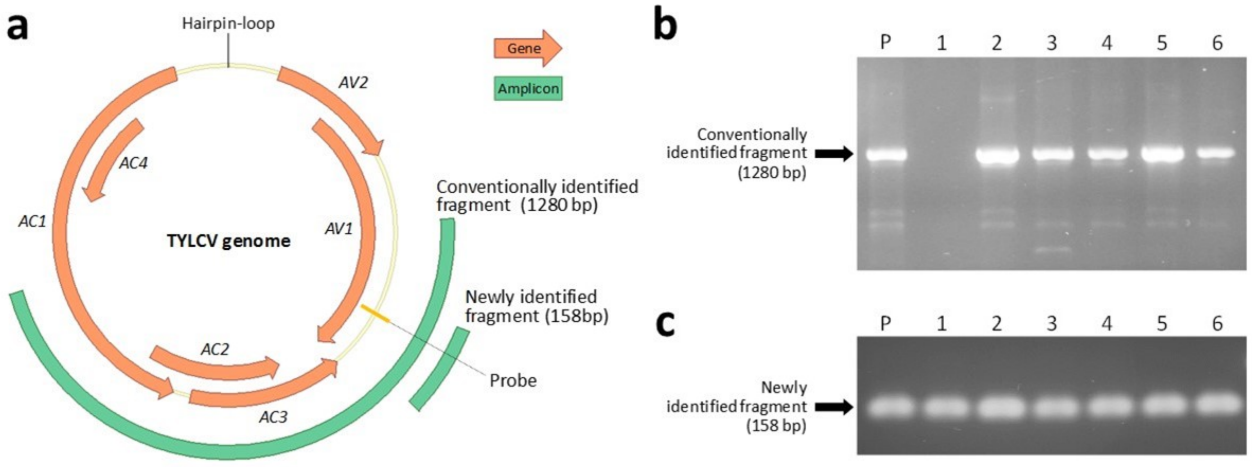

Supplementary Figure S2. Specificity test of TYLCV DNA marker. (**a**) Schematic diagram of TYLCV genome structure, and position of amplicons and AuNP probe. DNA detection of TYLCV in tomato using (**b**) conventionally identified primer set PAR1c715H/TH1978; the amplified length of the fragment is 1280 bp and (**c**) for the newly identified primer set 1/2R, the amplified length of the fragment is 158 bp. Lane P is TYLCV reference isolate 82-2-1; lanes 1-6 are various TYLCV-infected plants. Full-length gels are presented in Supplementary Figure S7.

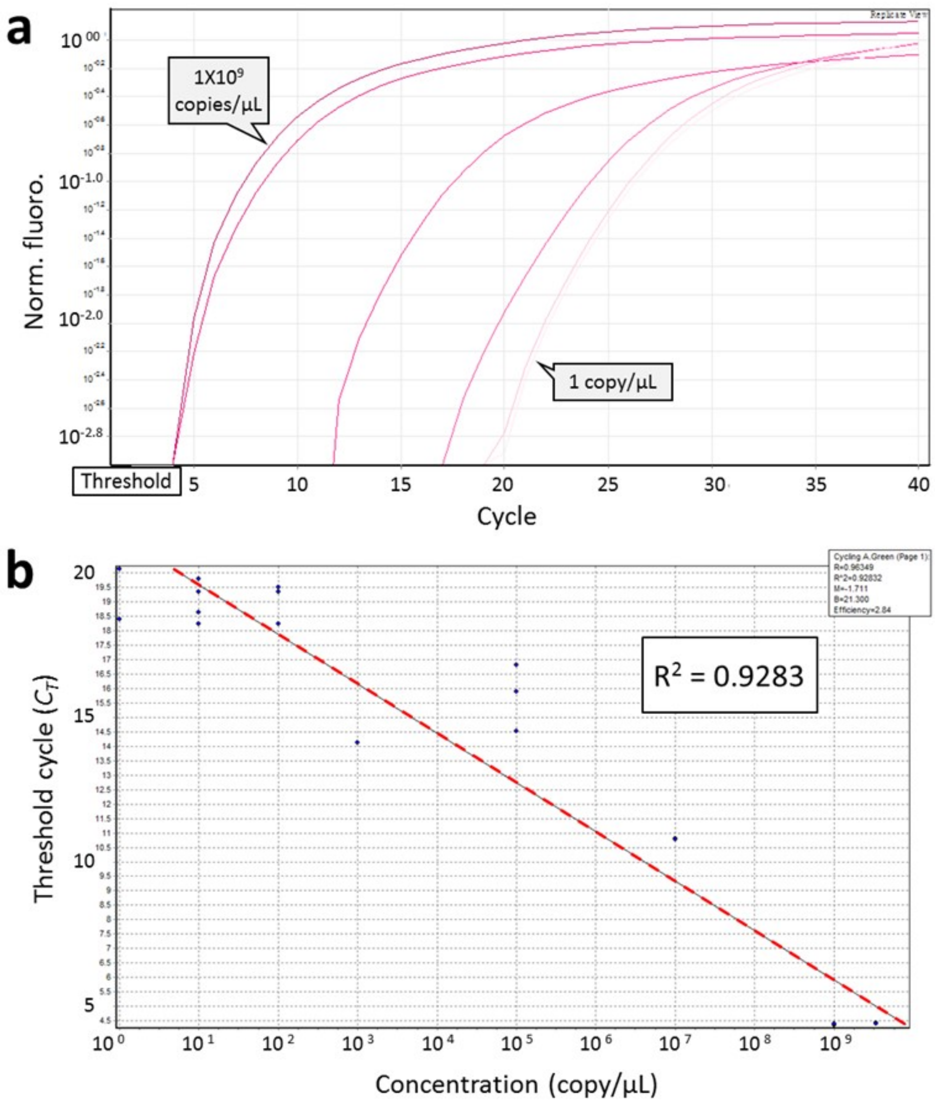

Supplementary Figure S3. Sensitivity test of TYLCV DNA diagnosis through qPCR. (**a**) Amplification curves of various template concentrations. (**b**) Plot of correlation between threshold cycle (C_T) and template concentration. One copy/μL of TYLCV is detectable after 20 cycles of a program, which is equivalent to 20 min

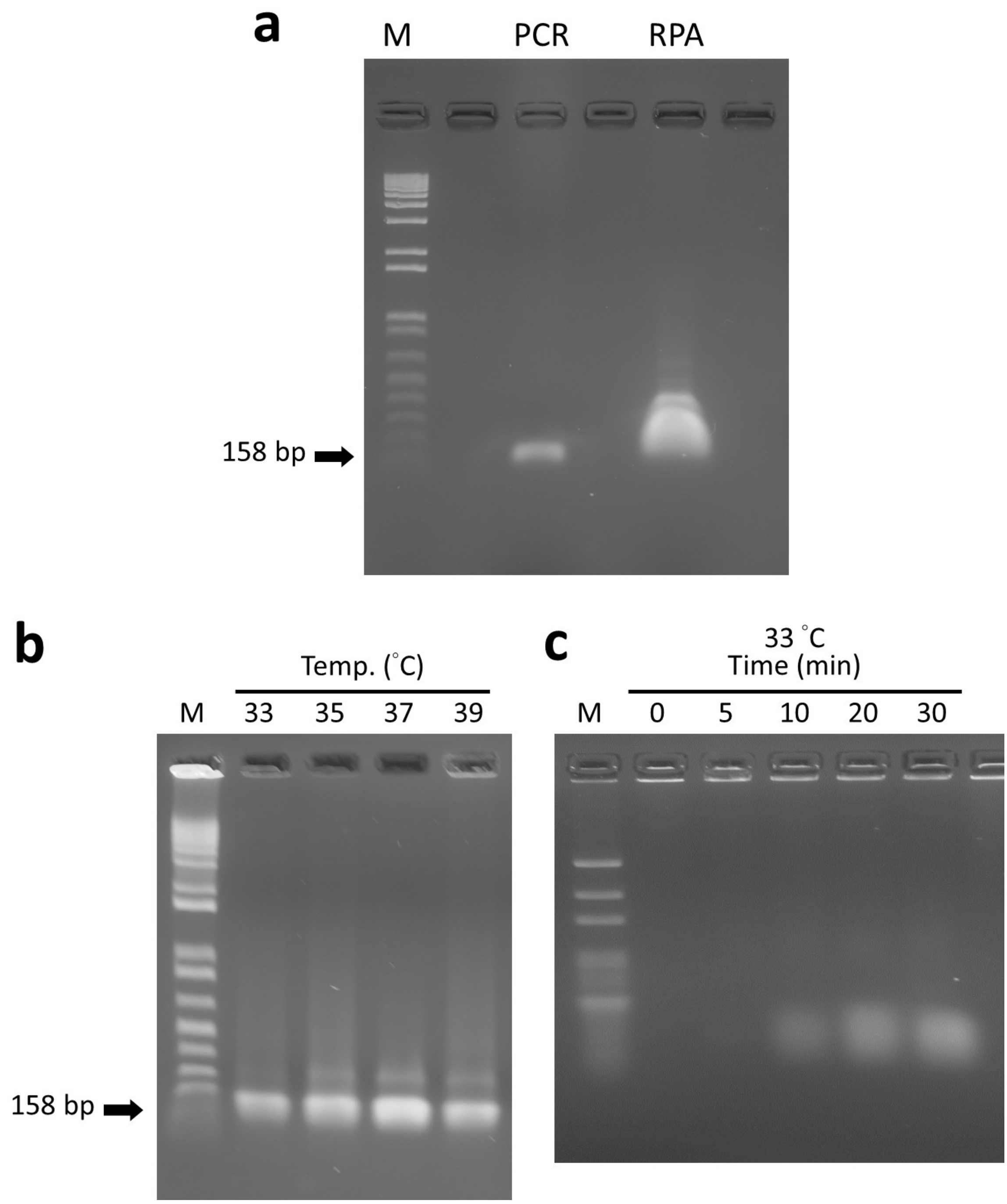

Supplementary Figure S4. Full-length gels of Figure 3. (**a**) Full-length gel of Figure 3a. (**b and c**) Full-length gels of Figure 3b. M, DNA ladder.

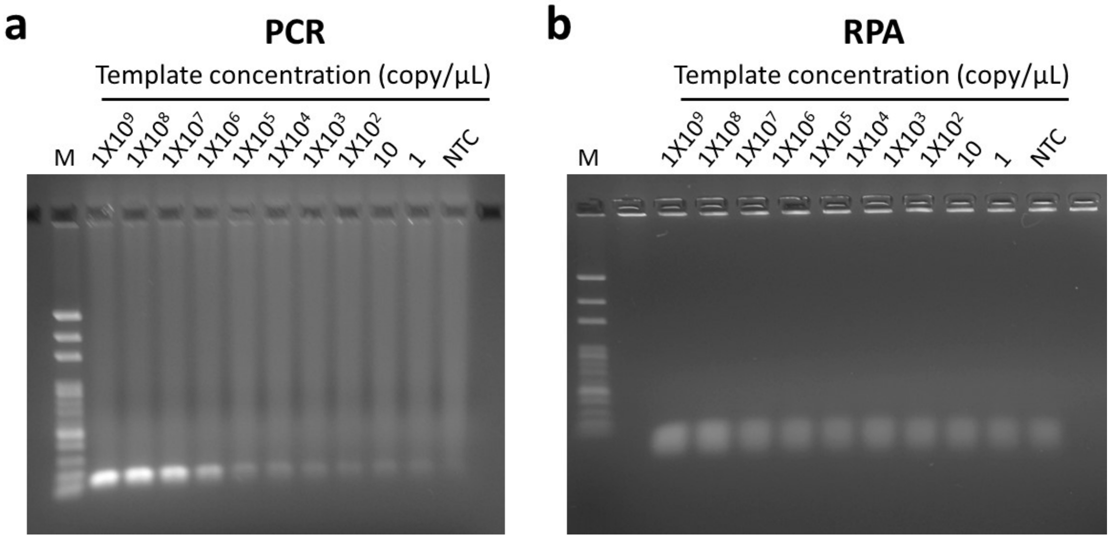

Supplementary Figure S5. Full-length gels of Figure 4. (**a**) Full-length gel of Figure 4a. (**b**) Full-length gel of Figure 4b. M, DNA ladder.

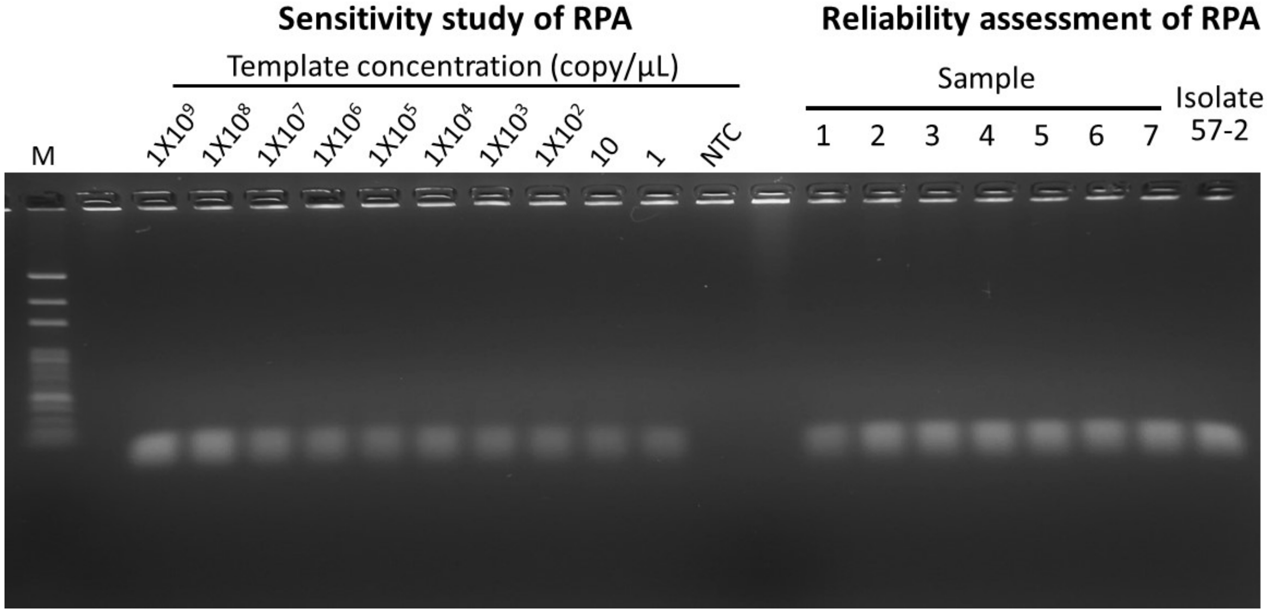

Supplementary Figure S6. Full-length gel of Figure 6 presents reliability assessment. M, DNA ladder.

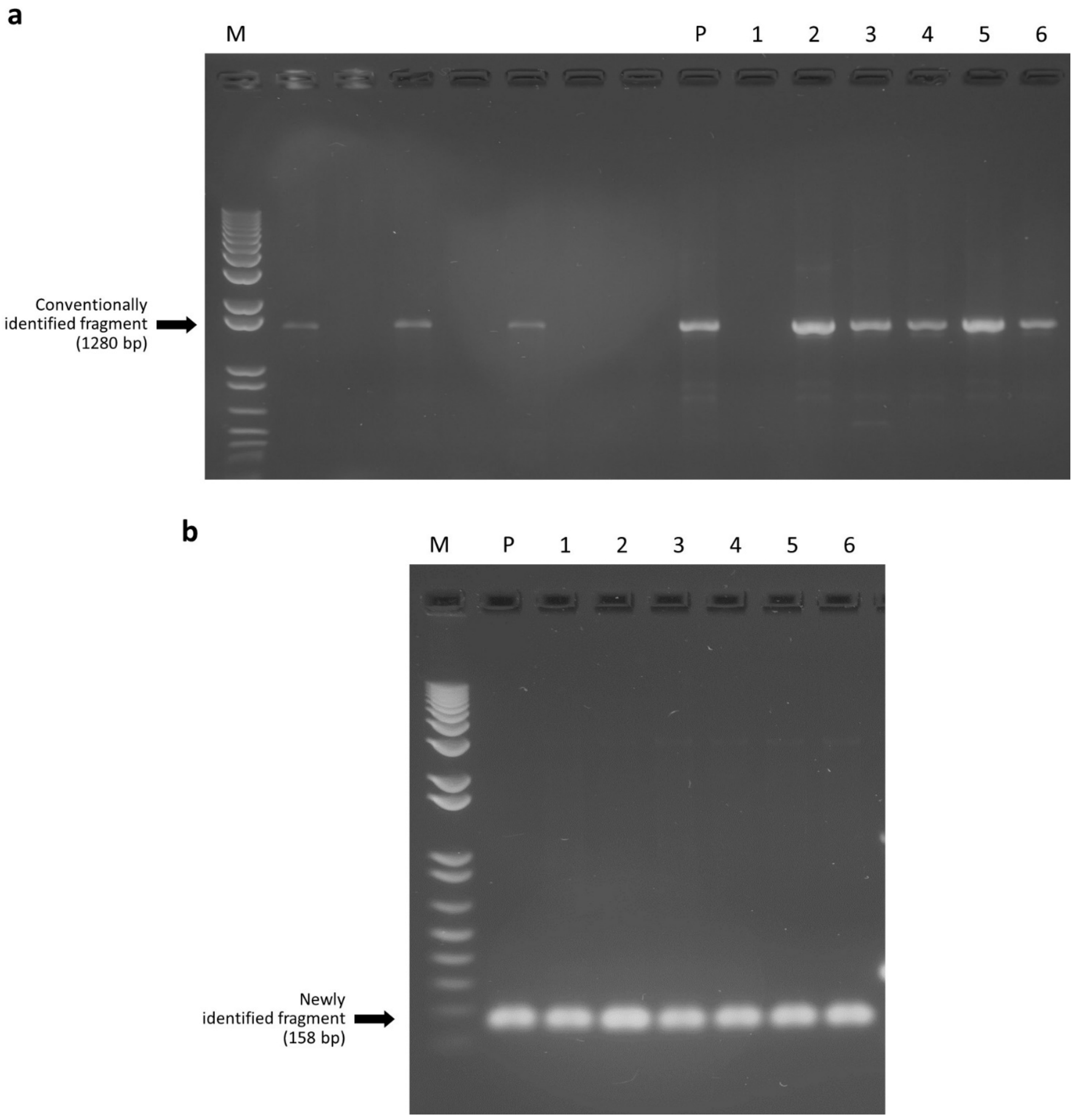

Supplementary Figure S7. Full-length gels of Supplementary Figure S2. (**a**) Full-length gel of Supplementary Figure S2b. (**b**) Full-length gel of Supplementary Figure S2c. M, DNA ladder.
